# Cardio-Oncology in Iran

**DOI:** 10.1016/j.jaccao.2021.09.011

**Published:** 2021-12-21

**Authors:** Azin Alizadehasl, Feridoun Noohi, Majid Maleki, Mohammad Mehdi Peyghambari, Niloufar Akbari Parsa, Amir Hossein Emami, Asadollah Moosavi, Mehrdad Hagh Azali, Hamidreza Pouraliakbar, Payam Azadeh

**Affiliations:** a Shaheed Rajaei Cardiovascular Medical and Research Center, Cardio-Oncology Research Center, Iran University of Medical Science, Tehran, Iran; b Shaheed Rajaei Cardiovascular Medical and Research Center, Iran University of Medical Science, Tehran, Iran; c Emam Khomeni Hispital, Tehran University of Medical Sciences, Tehran, Iran; d Hematology, Oncology and Stem Cell Transplantation Research Center, Tehran University of Medical Sciences, Tehran, Iran; e Emam Hossein Hospital, Shaheed Beheshti University of Medical Sciences, Tehran, Iran

**Figure:**
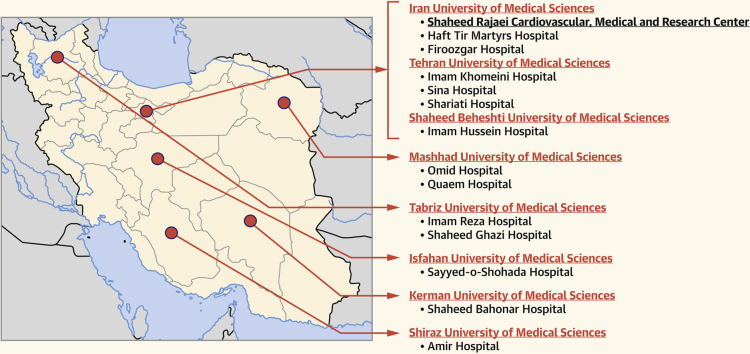

Iran is a developing country located in the Middle East, which has experienced rapid development caused by industrialization and modernity. The resulting lifestyle and environmental changes in recent years, along with improvements in life expectancy and the growing elderly population, are postulated to have an impact on cancer prevalence and the epidemiologic patterns of various types of malignancies (1).

In Iran, cancer is the second largest group of chronic noncommunicable diseases and the third most common cause of death after heart disease and motor vehicle accidents (2). The age-standardized incidence rates of cancers were 110 and 98 per 100,000 among male and female Iranians, respectively, and the estimated cancer mortality rates were 65 and 41.1 per 100,000, respectively (1). The 5 most common cancers (other than skin cancer) were stomach, prostate, lung, colorectal, and bladder in male Iranians and breast, colorectal, stomach, lung, and thyroid in female Iranians (3).

The main purpose of cardio-oncology is to consider aspects of both cardiology and oncology in a patient, creating a bridge between the 2 fields to make the best decisions regarding the prevention, monitoring, and treatment of the diseases. To accomplish this, it is crucial to establish a collaborative effort to achieve the best possible cancer and cardiovascular outcomes (4).

## Iran’s Health Care System

The health care system of Iran is organized at 3 levels: national, provincial, and city. At the national level, the Ministry of Health and Medical Education is the main coordinated headquarters of the health care and medical education system. At the provincial level, universities of medical sciences and health services are responsible for monitoring the activities of the health network and providing medical education and health research. Last, the health network of each city is considered the smallest independent unit that monitors the activities of the health care centers in the covered areas. All universities of medical sciences run many hospitals in different cities, including both general hospitals and those that specialize in a particular field. These educational hospitals train students in various medical fields (doctors, dentists, nurses, pharmacologists, midwives, among others) and are run by interns, residents, and fellows under the supervision of professors. These hospitals are public and have insurance contracts. However, it should be noted that, as in all countries, private hospitals have an important role in the country’s health system and in reducing the patient load.

Medical students in Iran complete a 7-year course to become general practitioners. They first work as general practitioners for 2 years. Then, after passing an examination and choosing the field of cardiology, they become cardiology assistants. After 4 years as cardiology assistants, they become cardiologists.

## Cardio-Oncology Clinics

The first cardio-oncology clinic in Iran was instituted about 4 years ago (2017) at Shaheed Rajaei Cardiovascular Medical and Research Center (the Cardiology Center of Iran University of Medical Sciences) in Tehran. To our knowledge, this was the first cardio-oncology clinic in the Middle East and functions as a center for visiting patients with cancer and who also have histories of cardiovascular disease or risk for cardiotoxicity during cancer therapy, as well as for patients affected by primary or metastatic tumors involving the heart. All patients with cancer are referred to this clinic before starting therapeutic agents for cancer and undergo careful assessment, including baseline cardiovascular state and cardiovascular risk factors. Then, all records are registered, and patients undergo risk stratification according to the data gathered.

All of our patients are enrolled in the multicenter Cardio-Oncology Toxicity Registry research database with the collaboration of partners from 13 other centers. All new patients with cancer who are referred to active oncologists in the project are registered in this program. Verbal and written consent is obtained from patients who agree to being enrolled in the program. After initial intake, a treatment plan for the patient is proposed. The patient forms detailing the history and plan are then submitted to us, either manually or electronically. At this center, the patient is visited by a physician and undergoes advanced echocardiography (or any other multimodality imaging needed). If necessary, medication is prescribed, and the final information on the form is completed by the physician. If necessary, primary prevention measures are prescribed in the form of lifestyle modification or medication therapy for their underlying cardiovascular conditions. The potential cardiovascular complications of various treatment strategies are discussed with the oncologist, and the best strategy is chosen for each patient. Depending on the treatment plan selected, the frequency of follow-up visits planned, and the potential development of treatment complications, the cardio-oncology team decides on stopping or modifying the cancer treatment strategy. Furthermore, all enrolled patients undergo long-term follow-up at this center, knowing that complications of cancer therapy may appear years after treatment. Currently, 2,000 patients are represented in our center’s registry.

The main purpose of this registry is to collect data about the epidemiologic factors affecting the Iranian population in cancer and heart disease. Risk factors, patient response to therapy, relapse, and factors affecting cardiac complications are collected, along with demographic and racial information for comparison with regional (ie, Middle East and Asia) and worldwide statistics. We believe that these data will be of benefit to the development of national guidelines and inform patient care in cardio-oncology.

Many specialists and health caregivers collaborate to form the cardio-oncology team. A cardio-oncologist and professor of echocardiography is the founder and head of the team. A group of heart failure and cardiac imaging specialists at our center and hematologists, radiation oncologists, and trained nurses at other centers are other members of the team. Furthermore, because of the occurrence of coronary artery and valvular complications of cancer therapeutics, which are often challenging, we benefit from the collaboration of experienced interventionalists and cardiovascular surgeons. For example, at this center, we implant chemotherapy ports for patients, manage complications such as broken ports, and perform transcatheter aortic valve replacement and the minimally invasive excision of cardiac tumors. Pathologists, nutritionists, primary care physicians, psychiatrists, and palliative care specialists also provide support for our cardio-oncology team.

**Figure 1:**
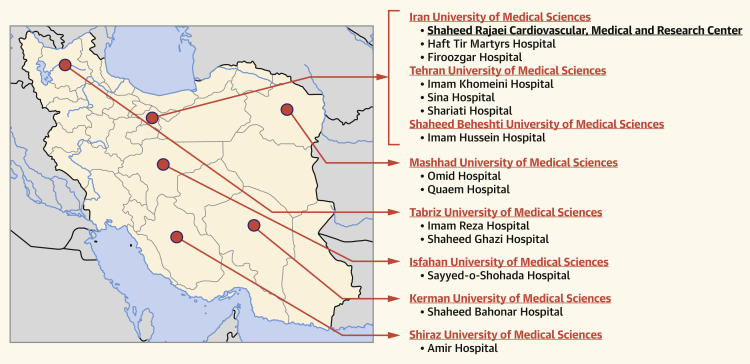
Centers Involved in the Cardio-Oncology Registry in Iran Shaheed Rajaei Cardiovascular Medical and Research Center is the referral hospital for cardio-oncology. The other hospitals are oncology (chemotherapy and radiotherapy) centers.

The main cardio-oncology clinic, which is the referral center, is located in Shaheed Rajaei Hospital, but as mentioned earlier, 13 other hospitals, which are oncology centers, are involved in the registry (6 hospitals from 3 different universities of medical sciences in the capital city, Tehran, and 7 other hospitals in Mashhad, Kerman, Shiraz, Isfahan, Tabriz, and Kerman). Although these 13 centers are oncology hospitals, they have active cardiologists and use the same system of documentation and follow-up for their patients. If any cardiac complications occur, the patient will be referred to our cardio-oncology clinic (Figure 1).

## Congresses, Seminars, and Webinars

One of the programs of the team is to organize numerous conferences and congresses with the participation of many experts to inform all physicians and health caretakers across the country about new advancements in this field. These events were also held as webinars during the coronavirus disease-2019 (COVID-19) pandemic. Furthermore, biweekly webinars are held at this center to introduce challenging cardio-oncology cases, with the active cooperation of cardiologists and oncologists as well as fellows and residents, which have received highly positive feedback.

## Cardio-Oncology Research Center

Research is the base of a cardio-oncology clinic. The cardio-oncology research center was opened in December 2019. Information for all patients is recorded at this center. Moreover, various clinical trials and research projects are advancing to promote our knowledge about this emerging field and propose the best therapeutic protocols and practical guidelines. The center has already published numerous papers and books.

## Education and Training

An important mission of the cardio-oncology unit is educating all residents and fellows in the field of cardiology and especially to train experts to spread this knowledge throughout the country, as we will need more specialists in the near future because of the growing prevalence of cancer survivors. The cardio-oncology training course started as a 6-month program, and this year, with the approval of the curriculum presented following several meetings in the Ministry of Health and Medical Education, cardio-oncology fellows will start an 18-month fellowship course at this center in the coming season. All cardiologists can choose to be cardio-oncologists and take the fellowship course in our center after passing the examination.

## COVID-19 pandemic

Furthermore, the consequences of each event on cardio-oncology patients are assessed and solutions are proposed, considering global and national conditions. During the COVID-19 pandemic, studies were carried out, and papers on the pandemic’s effects on cardio-oncology patients were published. In addition, guidelines on the COVID-19 pandemic and cardio-oncology patients were submitted to the Ministry of Health and Medical Education.

Fortunately, we can now say that all measures needed for prevention, therapy, follow-up, education, and research are currently active at this center, collectively forming a comprehensive field. Any efforts to help patients with cancer to lessen cardiac complications and extend their life expectancy and improve their quality of life will be valuable.

## Limitations and Challenges

Our greatest challenge in building cardio-oncology in Iran was its unfamiliarity to oncologists and cardiologists. We held many gatherings and seminars to introduce the wide scope of cardio-oncology, create acceptance of it as a new field of research and clinical practice, and explain how effective it can be to decrease patients’ symptoms, optimize their quality of life, decrease their morbidity and mortality, and increase their life expectancy. The results were incredible, with cardio-oncology now a well-appreciated field of medicine in Iran.

Despite all the measures and advances mentioned earlier, the emerging field of cardio-oncology in Iran is encountering several common challenges: 1) the cardio-oncology curriculum and guidelines must be approved so as to have comprehensive and unified protocols; 2) communication and interaction between oncologists and cardiologists must be strengthened throughout the country; and 3) financial resources are limited.

We hope that now that cardio-oncology has been approved as a fellowship course, more financial resources will be allocated for cardio-oncology educational, research, and clinical programs. At present, we have only 1 cardio-oncology center and many oncology centers involved in the program, but with the education of a new generation of fellows, many cardio-oncology clinics will very soon be founded in all of the provinces to spread this science all over the country.

## Funding Support and Author Disclosures

The authors have reported that they have no relationships relevant to the contents of this paper to disclose.
